# Altered Sexual Response-Related Functional Connectivity and Morphometric Changes Influenced by Sex Hormones across Menopausal Status

**DOI:** 10.3390/jcm13020387

**Published:** 2024-01-10

**Authors:** Chung Man Moon, Suk Hee Heo, Woong Yoon, Byung Hyun Baek, Sang Soo Shin, Seul Kee Kim, Yun Young Lee

**Affiliations:** 1 Research Institute of Medical Sciences, Chonnam National University, 264 Seoyang-ro, Hwasun-eup, Hwasun-gun 58128, Jeollanam-do, Republic of Korea; 2 Department of Radiology, Chonnam National University Hwasun Hospital, 322 Seoyang-ro, Hwasun-eup, Hwasun-gun 58128, Jeollanam-do, Republic of Korea; 3 Department of Radiology, Chonnam National University Medical School, 42 Jebong-ro, Dong-gu, Gwangju 61469, Gyeonggi-do, Republic of Korea; 4 Department of Radiology, Chonnam National University Hospital, 42 Jebong-ro, Dong-gu, Gwangju 61469, Gyeonggi-do, Republic of Korea

**Keywords:** menopause, sexual hormone, functional connectivity, gray matter volume, sexual arousal

## Abstract

Our study retrospectively investigated differential patterns of the functional connectivity (FC) of core brain regions synchronous with morphometric changes associated with sexual dysfunction in menopausal women, and their correlations with sexual hormones. Twenty-three premenopausal women (mean age: 41.52 ± 7.38 years) and 21 menopausal women (mean age: 55.52 ± 2.80 years) underwent sex hormone level measurements with high-resolution T1 and functional magnetic resonance imaging (MRI) during rest, neutral, and sexual arousal conditions. Analysis of covariance adjusted for age was used to compare the FC and gray matter (GM) volume between the two groups. Menopausal women showed lower GM volumes in the superior frontal gyrus (SFG), superior temporal pole, parahippocampal gyrus (PHG), hippocampus (Hip), amygdala (Amg), and cerebellum (Cb) compared to premenopausal women (*p* < 0.05). In addition, compared to premenopausal women, menopausal women showed decreased FC of seed regions involved in the SFG, frontal eye fields, and Amg, as well as target regions involved in the PHG, Hip, inferior frontal gyrus, Cb, and vermis (*p* < 0.005). Furthermore, the FC between the right Amg and right Cb and between the left Amg and right Cb during sexual arousal in both groups was positively correlated with total estrogen and estradiol levels, respectively (*p* < 0.01). The GM volume values in the right Amg and right Cb were positively correlated with total estrogen and estradiol levels (*p* < 0.05). Our study demonstrated an association between menopause-related differential FC and GM volume variations and fluctuating sex hormones. Our findings highlight that overlapping brain regions with functional alterations and morphometric changes are closely linked with menopausal symptom-related decreases in sexual arousal and hormone levels.

## 1. Introduction

Menopause is a natural and inevitable period of a woman’s aging process and refers to the permanent cessation of menstrual cyclicity, which is caused by a decline in ovarian follicular estrogen production [1]. A large proportion of menopausal women experience sexual dysfunction due to a lack of ovarian hormones, which can lead to functional changes in target organs, including low libido, difficulty with vaginal lubrication, and inability to climax [2]. Hormonal changes during menopause, along with psychological, sociocultural, physiological, interpersonal, and lifestyle factors, contribute to midlife sexual problems [3].

Over the past two decades, neuroimaging studies [2,4,5,6] using functional magnetic resonance imaging (fMRI) and positron emission tomography have demonstrated that brain centers are associated with visually evoked sexual arousal, including the limbic areas, comprising the amygdala (Amg), insula, anterior cingulate gyrus, thalamus, and hypothalamus. Although these studies have increased our understanding of the neuronal activity associated with sexual responses, they have primarily focused on identifying the distributed brain areas induced by various experimental design and activation paradigms. However, functional connectivity (FC) between the core brain areas is critical to further understand the neural network associated with sexual response. The use of task-based brain FC has enabled the identification of complex neuro-functional networks linked to corresponding task performance [7]. In this regard, FC between the core brain areas is critical to further understand the neural network because of its ability to provide the temporal dependency of anatomical brain region neural activation patterns, which is advantageous in developing standardized MR imaging-based neurological biomarkers for the accurate diagnosis and prediction of menopause [8,9].

A previous study [10] demonstrated that sex hormones act across the entire brain, engaging in neuroendocrine phenomena and establishing behavioral patterns, mood, and cognition. In particular, estrogen has profound effects on brain regions with a high density of estrogen receptors, which in turn can affect various functions regulated by those regions. Dysregulated estrogen signaling can impact cognitive and mood functions regulated by the prefrontal, hippocampal, Amg, and cingulate regions [11]. However, despite the importance of these effects, functional neuroimaging techniques have been limited to assess fundamental neuroendocrine physiological function in humans.

Moreover, estrogen enhances the structural integrity of brain tissue, inducing neuronal growth and exhibiting similar trophic effects. A few morphometric studies [12,13] have focused on the effects of estrogen therapy on postmenopausal women, which have demonstrated enhanced cognitive function and brain volume. However, to the best of our knowledge, these studies have not been comprehensively integrated with knowledge to uncover notable insights into how sex hormones impact both simultaneous functional connectivity (FC) and morphometric changes in the brain.

In this study, we evaluated the FC and morphometric changes during the sexual response between pre- and menopausal states, while accounting for the impact of sex hormones.

## 2. Materials and Methods

### 2.1. Ethics Approval

Ethical approval for this retrospective study was granted by the institutional review board (IRB) of Chonnam National University Hospital (CNUH) in accordance with the Declaration of Helsinki. The retrospective nature of this study led to the waiver of the requirement for written informed consent. However, it is important to note that each participant had previously submitted an informed consent form for their participation in a prior examination study [2]. All experimental protocols were executed in compliance with the applicable guidelines and regulations sanctioned by the IRB at CNUH.

### 2.2. Subjects

Twenty-three premenopausal women ranging in age from 23 to 51 years (mean age: 41.52 ± 7.38 years) and 21 menopausal women ranging in age from 49 to 59 years (mean age: 55.52 ± 2.80 years) were recruited. The enrollment criteria for menopausal women included the following [2]: (i) right-handedness; (ii) heterosexuality; (iii) menopause was diagnosed according to the Stages of Reproductive Aging Workshop (STRAW) + 10 staging system; (iv) no regular menstrual cycles in the previous year and follicle-stimulating hormone (FSH) level > 40 mIU/mL; and (v) no history of hormone or steroid treatment or oral contraceptive use in the month before the study. Premenopausal women were recruited based on the following criteria: (i) did not meet the diagnosis criterion of STRAW + 10; (ii) had a regular ovulation day based on the rhythm method; (iii) no history of perimenopause; and (iv) women without psychiatric and neurological illnesses.

Sexual function was evaluated using a validated Korean version of the female sexual function index (FSFI) questionnaire for six domains of desire, arousal, lubrication, orgasm, satisfaction, and pain [14].

### 2.3. Sex Hormone Measurements

All participants underwent sex hormone level testing, which included estradiol (E2), estriol (E3), luteinizing hormone (LH), total estrogen, FSH, sexual hormone binding globulin (SHBG), and free testosterone. Measurements were performed at 3–5 days after the onset of menstruation in the early follicular phase. Hormone testing was performed between 8:30 and 10:00 AM, which included blood collection, serum separation, and measurement of sex hormones by radioimmunoassay (Cobra 5010, Packard Instrument, Meriden, CT, USA) and chemiluminescent immunoassay (Bayer Healthcare, Chicago, IL, USA) [15].

### 2.4. Functional MRI Paradigm

During the fMRI scanning session, all women underwent the visual stimulation paradigm [16], which consisted of a rest period (cross fixation) for 30 s, a neutral period (natural documentary clips: forests, rivers, and mountains) for 30 s, a sexual stimulation period (erotic video clips: a heterosexual act including caressing, fellatio, and cunnilingus) for 540 s, and a rest period (cross fixation) for 30 s. Prior to the fMRI experiment, the erotic video and natural documentary clips were approved by 25 college students (mean age: 30.12 ± 3.06 years).

### 2.5. Magnetic Resonance Imaging Acquisition

All subjects were examined using a 3 Tesla Magnetom Trio MRI Scanner (Siemens Medical Solutions, Erlangen, Germany). The high resolution T1-weighted images were acquired using a three-dimensional magnetization prepared rapid gradient echo pulse sequence with a repetition time/echo time (TR/TE) = 1900 ms/2.35 ms, field of view (FOV) = 256 × 256 mm^2^, matrix size = 256 × 256, voxel size = 1 × 1 × 1 mm^3^, and slices = 176.

Functional images were obtained by a gradient-echo echo-planner imaging sequence with the following parameters: TR/TE = 2000/30 ms, flip angle = 90°, FOV = 22 × 22 cm^2^, matrix size = 64 × 64, and number of excitations = 1. Each slice image was acquired from the axial plane parallel to the anterior commissure-posterior commissure (AC-PC) line.

### 2.6. Data Preprocessing

#### 2.6.1. Whole Brain Volumetry

The high resolution T1-weighted images were post-processed using the statistical parametric mapping program (SPM 12; Wellcome Department of Imaging Neuroscience Group, University College London, London, UK) with diffeomorphic anatomical registration through an exponentiated Lie algebra-based voxel-based morphometry analysis to assess differences in the volume of gray matter (GM) between the groups. As reported previously [17], the T1 images of the all participants were aligned with an AC-PC line. After image non-uniformity correction to remove smoothly varying modulations of image intensity, these images were segmented into GM, white matter (WM), and cerebrospinal fluid (CSF) using tissue probability maps. Then, these images were normalized to the International Consortium of Brain Mapping space template for the East Asian brain type. The mean template for GM was generated from individual GM images. All images were spatially normalized to the Montreal Neurological Institute template and subsequently separated into GM images. An 8-mm full width at the half-maximum (FWHM) isotropic Gaussian kernel was used to smooth the GM images. The total intracranial volume (TIV) was calculated as the sum of the GM, WM, and CSF volumes in each woman. Analysis of covariance (ANCOVA) adjusted for age was used to compare the GM volume between the two groups.

#### 2.6.2. Functional Connectivity Analysis

The functional images were preprocessed using SPM 12 and the CONN toolbox software version 18a (Neuroimaging Informatics Tools and Resources Clearinghouse), both of which were implemented in MATLAB (MathWorks, Inc., Natick, MA, USA). The default preprocessing steps of CONN were selected as follows: slice-timing correction, realignment (subject-motion threshold: 2 mm), and normalization to the standard echo-planar image template in the Montreal Institute of Neuroscience space. Then, these images were smoothed with an 8 mm FWHM isotropic Gaussian kernel, and a band pass filter of 0.01–0.1 Hz was used to temporally filter the data. To minimize the effect of motion and physiological noise signals and other artifacts, an anatomical component-based noise correction approach (CompCor) was used to remove unwanted noises from blood-oxygenation-level-dependent signals [18].

FC measures were computed between seed areas for the region of interest to region of interest (ROI-to-ROI) analysis. All ROIs were drawn from Automated Anatomical Labeling (AAL), including 90 cortex ROIs and 26 cerebellar ROIs [19]. We compared the FC between premenopausal and menopausal women using ANCOVA adjusting for age (uncorrected at *p* < 0.005) in rest, neutral, and sexual arousal periods.

### 2.7. Statistical Analysis

The difference in characteristics between the two groups was analyzed by independent two-sample *t*-test using the statistical package for the social sciences (SPSS ver.20; Chicago, IL, USA). Pearson’s correlation analysis was performed to analyze the relationship between the brain FC, GM volume, and sex hormones.

## 3. Results

### 3.1. Demographic Characteristics

The comparison of the sex hormone concentrations in premenopausal and menopausal women is shown in Table 1. The two groups showed significant differences in the average levels of estradiol (*p* < 0.001), total estrogen (*p* < 0.001), FSH (*p* < 0.001), LH (*p* < 0.001), SHBG (*p* < 0.001), and free testosterone (*p* = 0.020), whereas there were no significant differences in estriol (*p* = 0.519).

The average full-scale score of FSFI was 25.61 ± 3.23 and 16.48 ± 2.03 in premenopausal and menopausal women (*p* < 0.001), respectively, with significant differences in the scores for arousal, lubrication, orgasm, satisfaction, and pain (all scores, *p* < 0.001), but not for desire (*p* = 0.197) (Table 1).

### 3.2. Localized Differences in Brain Volume

The TIVs in the two groups are summarized in Table 2. The GM volumes in premenopausal and menopausal women were 629.20 ± 50.44 mL and 582.44 ± 44.23 mL (*p* = 0.056), the WM volumes were 501.33 ± 55.36 mL and 460.88 ± 51.07 mL (*p* = 0.150), the CSF volumes were 451.37 ± 102.83 mL and 490.02 ± 109.87 mL (*p* = 0.718), and the TIVs were 1581.90 ± 137.09 mL and 1533.34 ± 155.62 mL (*p* = 0.155), respectively. However, none of the tissue volumes and TIVs were significantly different between groups.

**Figure 1:**
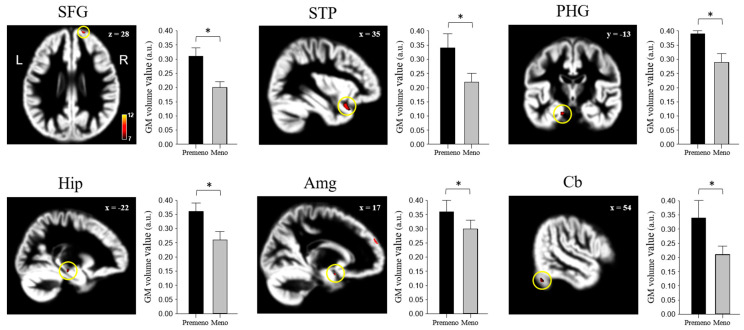
Brain areas showed lower gray matter volumes in menopausal women than in premenopausal women by analysis of covariance after age adjustment. The color-coded pixels were scaled to a range (t-value) greater than the cut-off threshold. * Statistically significant difference at *p* < 0.05. SFG: superior frontal gyrus, STP: superior temporal pole, PHG: parahippocampal gyrus, Hip: hippocampus, Amg: amygdala, Cb: cerebellum, Premeno: premenopause, Meno: menopause, L: Left, and R: Right.

Menopausal women showed lower GM volumes in the superior frontal gyrus (SFG), superior temporal pole (STP), parahippocampal gyrus (PHG), hippocampus (Hip), Amg, and cerebellum (Cb) than premenopausal women (Figure 1 and Table 3) (family-wise error, *p* < 0.05), while there were no larger GM volumes in the menopausal women.

### 3.3. Differences in FC

**Figure 2:**
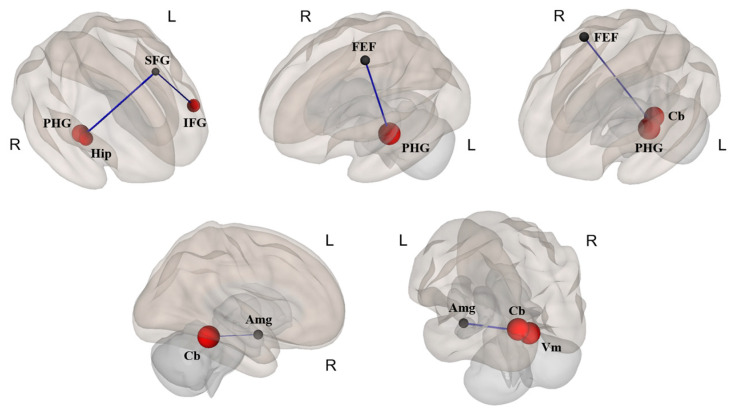
Altered ROI-to-ROI functional connectivity (FC) of networks in the premenopausal vs. menopausal group, showing decreased FC in all seed-target regions in the menopausal group. ROI: region of interest, SFG: superior frontal gyrus, PHG: parahippocampal gyrus, Hip: hippocampus, IFG: inferior frontal gyrus, FEF: frontal eye fields, Cb: cerebellum, Amg: amygdala, Vm: vermis, L: Left, and R: Right.

**Figure 3:**
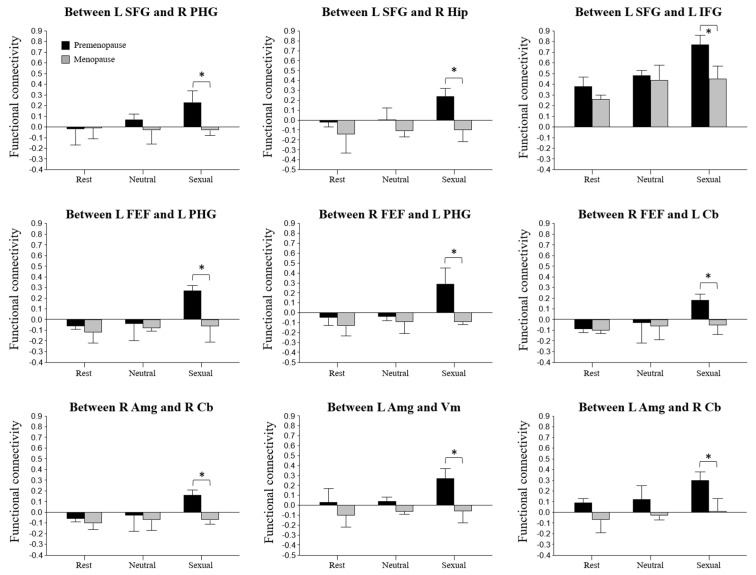
Comparison of functional connectivity values between the two groups during rest, neutral, and sexual arousal periods. * Statistically significant difference at *p* < 0.05. L: left and R: right.

The results of FC using ROI-to-ROI analysis between the two groups are shown in Figure 2 and Figure 3 and Table 4. Compared to premenopausal women, menopausal women showed decreased FC of seed regions involved in the SFG, frontal eye fields (FEF), and Amg, as well as the target regions involved in the PHG, Hip, inferior frontal gyrus (IFG), Cb, and vermis (Vm), as detailed in Table 4. However, the brain ROIs of the menopausal group showed no increase in FC.

### 3.4. Correlation of Sex Hormone Levels with FC and Localized GM Volume

**Figure 4:**
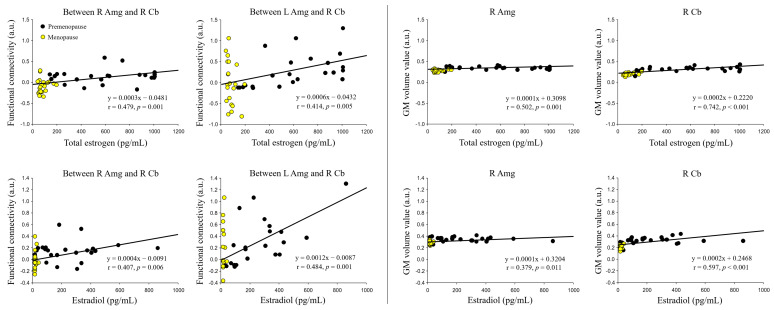
Positive correlations between total estrogen and estradiol levels and changes in functional connectivity and gray matter volume value during sexual arousal in a combined group of premenopausal and menopausal women.

As shown in Figure 4, the FC between the right Amg and right Cb (with total estrogen: r = 0.479, *p* = 0.001; estradiol: r = 0.407, *p* = 0.001) and between the left Amg and right Cb (with total estrogen: r = 0.414, *p* = 0.005; estradiol: r = 0.484, *p* = 0.001) during sexual arousal in the combined group of premenopausal and menopausal women was positively correlated with total estrogen and estradiol levels, respectively.

In addition, the GM volume values in the right Amg (with total estrogen: r = 0.502, *p* = 0.001; estradiol: r = 0.379, *p* = 0.011) and right Cb (with total estrogen: r = 0.742, *p* < 0.001; estradiol: r = 0.597, *p* < 0.001) were positively correlated with total estrogen and estradiol levels, respectively. However, there were no significant correlations between other brain FC regions and GM volumes.

## 4. Discussion

Menopausal symptoms are associated with disruptions in multiple hormone-regulated systems [20]. Sex hormones have been widely recognized for their crucial roles in procreation and the development of sexual maturity. However, a recent neuroendocrinology study [21] has also demonstrated their roles in brain plasticity. In addition, the fluctuations in serum hormones, especially estrogens, are known to affect the function and structure of the central nervous system through a network of hormone receptors [20]. Therefore, it is of clinical importance to study brain function alterations and to explore the association of serum hormone levels with brain functional and morphometric changes in menopausal women.

In the current study, the concentrations of sex hormones, including estradiol, estriol, total estrogen, FSH, LH, SHBG, and free testosterone, were measured to provide a general evaluation of endocrine status in the enrolled participants. The results indicated that the concentrations of the seven hormones were all within the normal range for both the premenopausal and menopausal groups. However, menopausal women experienced increased levels of FSH and LH and decreased levels of estradiol, total estrogen, SHBG, and free testosterone compared to premenopausal women. As supported by findings in animal studies [22,23], estrogen offers neurotrophic support, possibly through synergistic interactions with the cholinergic system, and it helps to regulate acetylcholine release. Estradiol, a type of estrogen, plays a crucial role in various physiological processes, including cognitive function, mood regulation, and learning and memory [24]. Estradiol is synthesized in the ovary, whereas FSH and LH are released by the pituitary gland [25]. During the menopause, decreased ovarian function results in decreased estradiol release [26]. As a result, the pituitary gland releases more FSH and LH to stimulate the ovary to produce steroids. Our results revealed that only estriol (*p* = 0.519) was not significantly different between the two groups.

Sexual function status was also assessed in the enrolled subjects. With regard to self-assessment of the six domain-based sexual functions in FSFI, the premenopausal and postmenopausal women scored 25.61 ± 3.23 and 16.48 ± 2.03 out of 36 points, respectively, meaning that the menopausal women were assumed to be physiologically and physically sexually dysfunctional, according to the threshold (<25 points).

In the present study, together with brain morphometry, FC was used to assess functional changes in brain connectivity related to sexual function in menopausal women while viewing the erotic video clip, and correlation analysis was used to evaluate the relationship of serum sex hormone levels with aberrant FCs and GM volume changes due to the integrity of the hormonal milieu being closely related to sexual function [27]. Our results demonstrated that certain regions related to sexual function showed FC and morphometric changes in menopausal women, and that aberrant sex hormone levels may account for the FC abnormalities and GM volume alterations.

Decreased FC of the SFG and FEF connected with the PHG and Hip, respectively, was found in menopausal women compared to premenopausal women. The SFG, which includes the supplementary motor area, is implicated in the motor control of sexual behavior and is involved in cognitive function and working memory [28]. The decreased spontaneous brain activity in the SFG in menopausal women could be assumed to indicate that menopausal women had a greater chance of experiencing decreased cognitive function and decreased working memory than premenopausal women. As such, this finding provides a potential explanation for sexual behavior problems with a decrease in cognitive functioning during menopause. In addition, PHG activation has been reported to be involved in women’s orgasms [29], which may thus be related to women’s sexual arousal. Compared to menopausal women who were estrogen users, menopausal women who were non-estrogen users showed significantly lower metabolism in the Hip and PHG over a 2-year observation period [30]. In addition, compared to premenopausal women, menopausal women showed lower activity in the PHG and Hip, which is implicated in sexual arousal in women [15]. It has been suggested that decreased activation areas in menopausal women are related to decreased sexual arousal, although the dorsal attention network, mainly composed of the FEF and the intraparietal sulcus, is an important mediator of goal-directed attentional processing. The FEF identified in this study has diverse functions spanning from perception to eye movements and the interactions between them [31]. Therefore, the decreased FC in the SFG and FEF connected with the PHG and Hip may be the cause of the difficulties facing sexual arousal experienced by menopausal women, reflective of a high prevalence of sexual and cognitive dysfunction in women during menopause.

Interestingly, regarding the FC between the Amg and Cb, menopausal women showed lower FC in the Amg than premenopausal women. The Amg has a crucial role in the mediation of sexual behavior in animals, and the Amg activation noted here is related to the emotional response processes through which the erotic stimuli are evaluated as sexual incentives [15]. That is, in menopausal women, the Amg could be associated with a menopause-related decrease in sexual arousal. Additionally, FC changes in the Amg were positively correlated with the levels of total estrogen and estradiol in premenopause and menopause groups in this study. It is speculated that decreased activity of the Amg in menopausal women is due to a lack of estrogen after menopause. Furthermore, a prospective observational study [32] reported that the decline in sexual function at menopause relates more to decreasing estradiol levels than to androgen levels. Such findings support the notion that the lower FC of the Amg in menopausal women is menopause-related. As a major site of sex steroid hormone action, the developing Cb exhibits de novo synthesis of estrogen and progesterone, and estrogen influences the formation of dendritic spines and synapses through the regulation of microglia [33]. The adult Cb exhibits abundant expression of estrogen receptors [33]. It has been suggested that sex hormones not only influence the formation of cerebellar neuronal circuitry during neonatal development but also modulate cerebellar functioning later in life. Taken together, these results suggest that estradiol is associated with the FC and network topology of the Cb, providing insight into the relationship between sex hormones and the intrinsic dynamics of the Cb. Although the mechanisms driving the unique effects of these hormones in the Cb have yet to be characterized, our findings imply that estradiol may synchronously decrease connectivity within the Cb. The positive correlation of FC values in the Amg and Cb with total estrogen and estradiol levels suggests that certain sexual hormones affect specific brain connectivities and volume changes related to sexual function and the brain’s sexual network. Consequently, these findings can be considered as important factors and early indicators of menopausal status.

Regarding the morphometric findings, postmenopausal women exhibited decreased SFG, STP, PHG, Hip, Amg, and Cb volumes compared to premenopausal women. Notably, both morphometric and FC changes were observed in the left PHG, right Cb, and right Amg. Overall, our results suggest that decreased GM volume and variations in FC associated with the PHG, Amg, and Cb are important morphometric and functional menopause-related symptoms, which could be associated with cell loss and/or cell shrinkage related to hormone loss [34]. Moreover, we found a positive correlation of the GM volume of the Amg and Cb with premenopausal and menopausal periods, which is likely due to the decrease in total estrogen following menopause and helps to explain the underlying total estrogen- and estradiol-related morphometric changes following menopause.

The current study has several limitations. First, we did not perform objective physiological or psychological testing of sexual functioning. Second, this study did not evaluate estrone (E1), despite its prevalence in postmenopausal women. It is advisable to include an assessment of estrone in future studies. Third, in consideration of the potential influence of hormone therapy and contraceptive use on the brain connectivity and structure of menopausal women, the inclusion criteria stipulated no history of hormone or steroid treatment, nor oral contraceptive use in the month preceding the study. Fourth, further studies should investigate whether other personal factors (e.g., depression, emotional status, marital characteristics, religion, and personality) may affect functional and morphological measurements.

## 5. Conclusions

Our study demonstrated an association between menopause-related differential FC and GM volume variations and fluctuating sex hormones. Our findings have significant implications for understanding the relationship between specific brain regions, such as the Amg and Cb, which exhibit both functional and morphometric changes, and the decline in sexual arousal and hormonal levels associated with menopausal symptoms.

## Figures and Tables

**Table 1:** Characteristics of premenopausal and menopausal women.

	Premenopausal Women (n = 23)	Menopausal Women (n = 21)	*p*-Value *
Age (year)	41.52 ± 7.38	55.52 ± 2.80	<0.001
Period after menopause (year)	−	6.00 ± 2.63	
Sex hormone levels			
Estradiol (E2) ^a^ (pg/mL)	244.98 ± 204.41	13.31 ± 6.45	<0.001
Estriol (E3) ^b^ (pg/mL)	2.74 ± 1.66	2.44 ± 1.40	0.519
Total estrogen ^c^ (pg/mL)	597.30 ± 312.69	77.97 ± 41.15	<0.001
Follicle-stimulating hormone ^d^ (FSH, mlU/mL)	6.63 ± 3.82	64.29 ± 20.41	<0.001
Luteinizing hormone ^e^ (LH, mlU/mL)	16.17 ± 15.47	35.50 ± 10.23	<0.001
Sexual hormone binding globulin ^f^ (SHBG, nmol/L)	99.81 ± 31.47	69.87 ± 17.13	<0.001
Free testosterone ^g^ (pg/mL)	0.46 ± 0.34	0.27 ± 0.17	0.020
Female sexual function index ^†^			
Desire	2.91 ± 1.00	2.51 ± 1.03	0.197
Arousal	4.08 ± 1.02	2.80 ± 1.07	<0.001
Lubrication	4.79 ± 1.37	2.81 ± 1.04	<0.001
Orgasm	4.56 ± 1.20	2.84 ± 1.05	<0.001
Satisfaction	4.37 ± 1.28	2.86 ± 1.18	<0.001
Pain	4.91 ± 1.20	2.67 ± 1.06	<0.001
Full scale score	25.61 ± 3.23	16.48 ± 2.03	<0.001

Data are presented as the mean ± SD. * The difference between the two groups was analyzed using an independent two-sample *t*-test. Reference ranges for sex hormones in premenopausal and menopausal women: ^a^ premenopausal women, 11–526 pg/mL; menopausal women, less than 37 pg/mL; ^b^ pregnant women, 38–460 ng/mL at 28–40 weeks; ^c^ premenopausal women, more than 61 pg/mL; menopausal women, less than 60 pg/mL; ^d^ premenopausal women, 1.5–33.4 mIU/mL; menopausal women, 23–116.3 mIU/mL; ^e^ premenopausal women, 0.5–73.6 mIU/mL; menopausal women, 15.9–54.0 mIU/mL; ^f^ women, 16–120 nmol/L; men, 10–73 nmol/L; ^g^ 20–39 years old, 0.06–2.5 pg/mL; 40–59 years old, 0.04–2.0 pg/mL. ^†^ Full marks of each domain is 6.

**Table 2:** Comparison of the brain volumes between the two groups by analysis of covariance with covariates of age.

Tissue	Premenopausal Women	Menopausal Women	*p*-Value
Gary matter (mm^3^)	629.20 ± 50.44	582.44 ± 44.23	0.056
White matter (mm^3^)	501.33 ± 55.36	460.88 ± 51.07	0.150
Cerebrospinal fluid (mm^3^)	451.37 ± 102.83	490.02 ± 109.87	0.718
Total volume (mm^3^)	1581.90 ± 137.09	1533.34 ± 155.62	0.155

**Table 3:** Lower gray matter volumes in menopausal women compared to premenopausal women: analysis of covariance adjusted for age (FWE-corrected *p* < 0.05).

Brain Area	MNI Coordinates			Voxels	*t*-Value
	X	Y	Z		
Superior frontal gyrus (SFG)	18	60	28	114	8.70
Superior temporal pole (STP)	35	16	−23	1371	10.10
Parahippocampal gyrus (PHG)	−18	−13	−26	165	9.70
Hippocampus (Hip)	−22	−22	−12	101	8.84
Amygdala (Amg)	17	4	−15	86	8.49
Cerebellum (Cb)	54	−63	−33	192	7.59

FEW: family-wise error; MNI: Montreal Neurological Institute.

**Table 4:** Decreased ROI-to-ROI functional connectivities for an individual seed region during sexual arousal in the menopausal group compared to premenopausal group (uncorrected *p* < 0.005).

Brain Area		MNI Coordinates			*t*-Value	*p*-Value
		X	Y	Z		
Seed region: Superior frontal gyrus (SFG)		−12	56	28		
Target region:	Parahippocampal gyrus (PHG)	24	−26	−16	3.76	0.0005
	Hippocampus (Hip)	29	−16	−14	2.98	0.0048
	Inferior frontal gyrus (IFG)	−51	26	2	2.72	0.0048
Seed region: Frontal eye fields (FEF)		−27	−9	64		
Target region:	Parahippocampal gyrus (PHG)	−20	−7	−29	2.97	0.0050
Seed region: Frontal eye fields (FEF)		30	−6	64		
Target region:	Parahippocampal gyrus (PHG)	−22	−10	−28	3.02	0.0043
	Cerebellum (Cb)	−26	−30	−30	2.74	0.0046
Seed region: Amygdala (Amg)		26	1	−15		
Target region:	Cerebellum (Cb)	27	−29	−25	3.45	0.0013
Seed region: Amygdala (Amg)		−25	−1	−18		
Target region:	Vermis (Vm)	2	−52	−20	3.24	0.0023
	Cerebellum (Cb)	28	−28	−29	3.21	0.0026

ROI: region of interest.

## Data Availability

The data samples are available upon request from the corresponding author. However, the data are not publicly available due to ethical or privacy restrictions.
